# Supplementary material for: Sensei: how many samples to tell a change in cell type abundance?
Source: BMC Bioinformatics. 2022 Jan 4;23:2. doi: 10.1186/s12859-021-04526-5 (PMC8728970; doi:10.1186/s12859-021-04526-5)
Supplement: Supplementary file 1 — Additional file 1: Figure S1–S13 and Supplementary Text. [file 12859_2021_4526_MOESM1_ESM.docx]

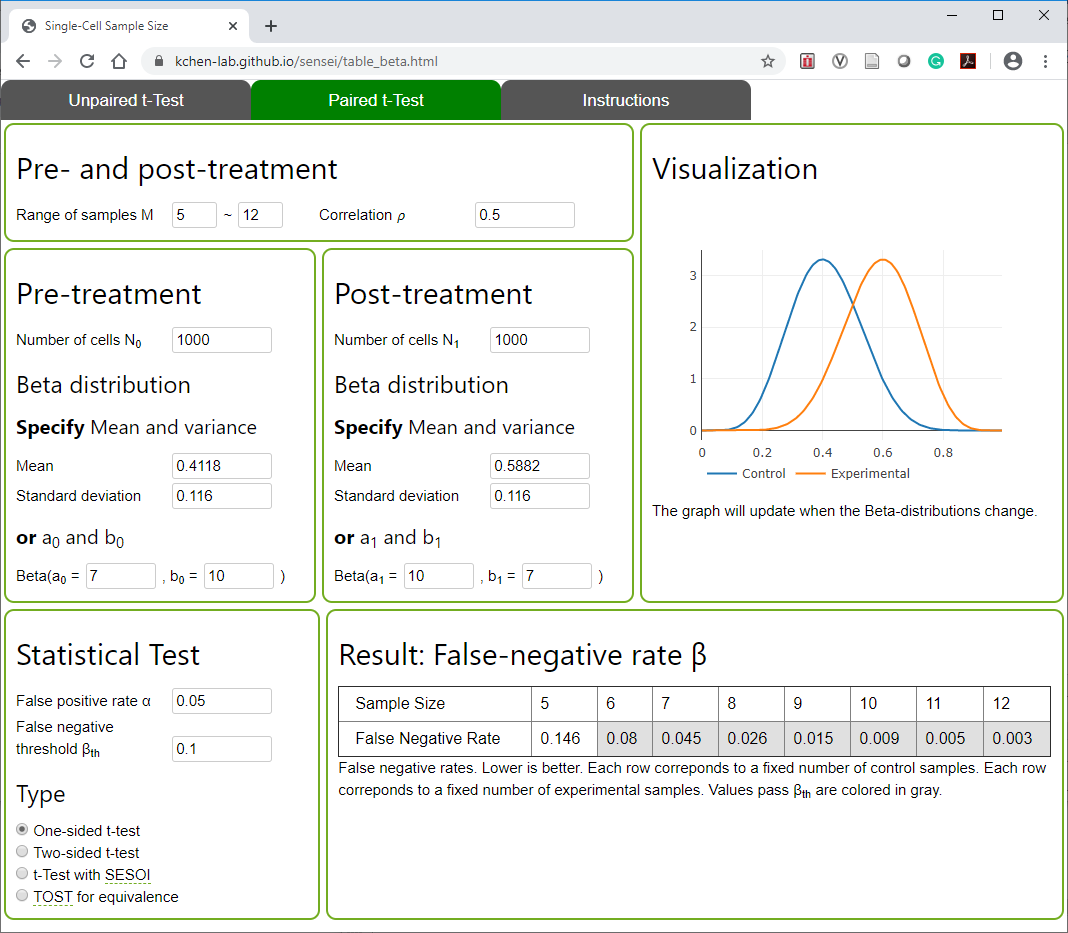


**Figure S1**. Screenshot of Sensei for paired samples.

**Figure S2.** The empirical distribution of the T-cell abundance in tumor group and juxtatumoral group, respectively.


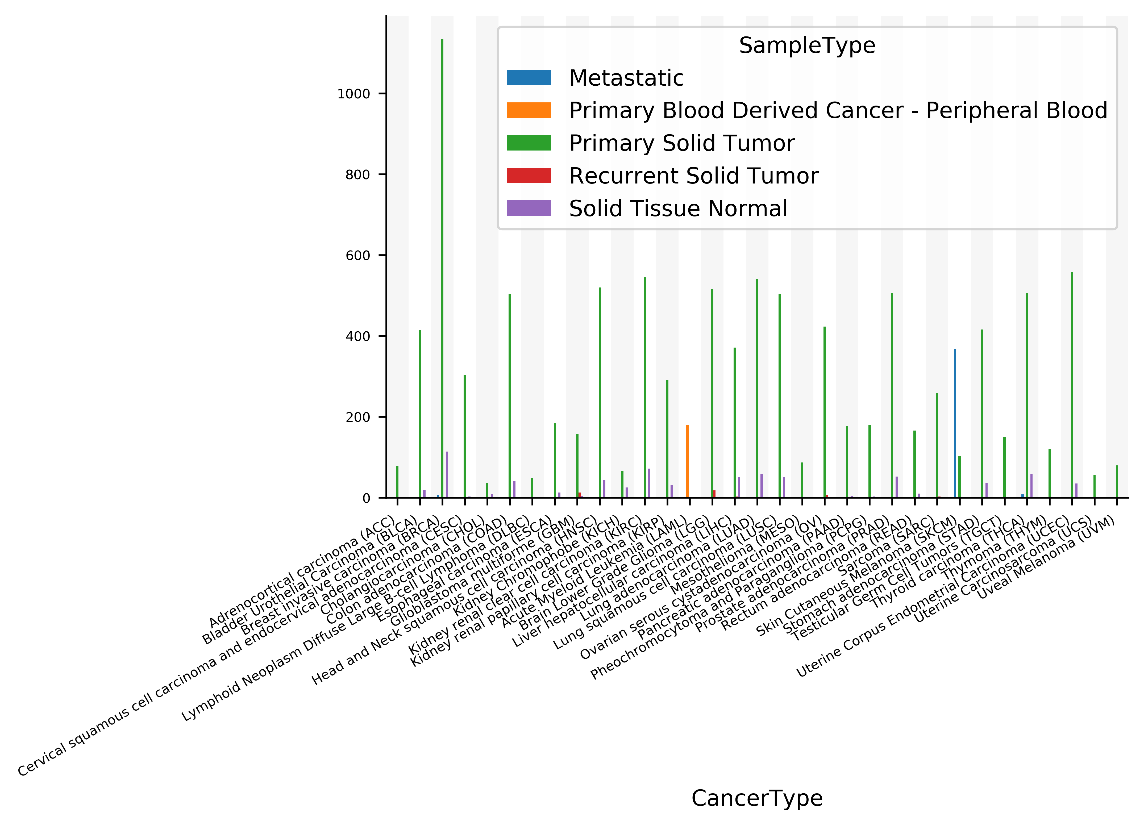


**Figure S3.** Number of samples available in TCGA for each sample type in each cancer type.


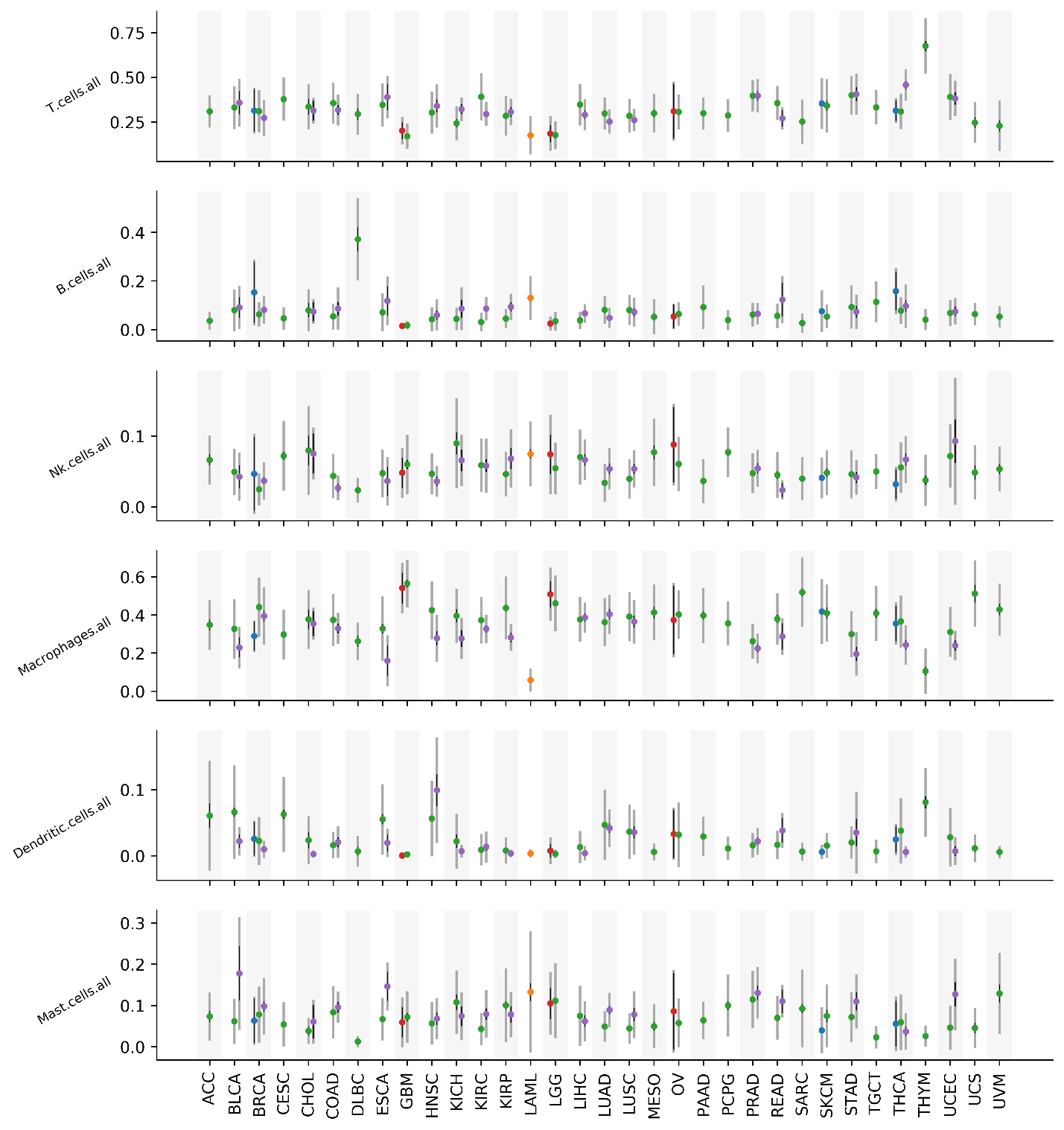


**Figure S4.** Mean (dot), 95% confidence interval of mean (thin black line), and standard deviation (wide gray line) of the abundance of each major immune cell types in the deconvolved TCGA data. Color correspond to sample type with the same scheme in Figure S3.


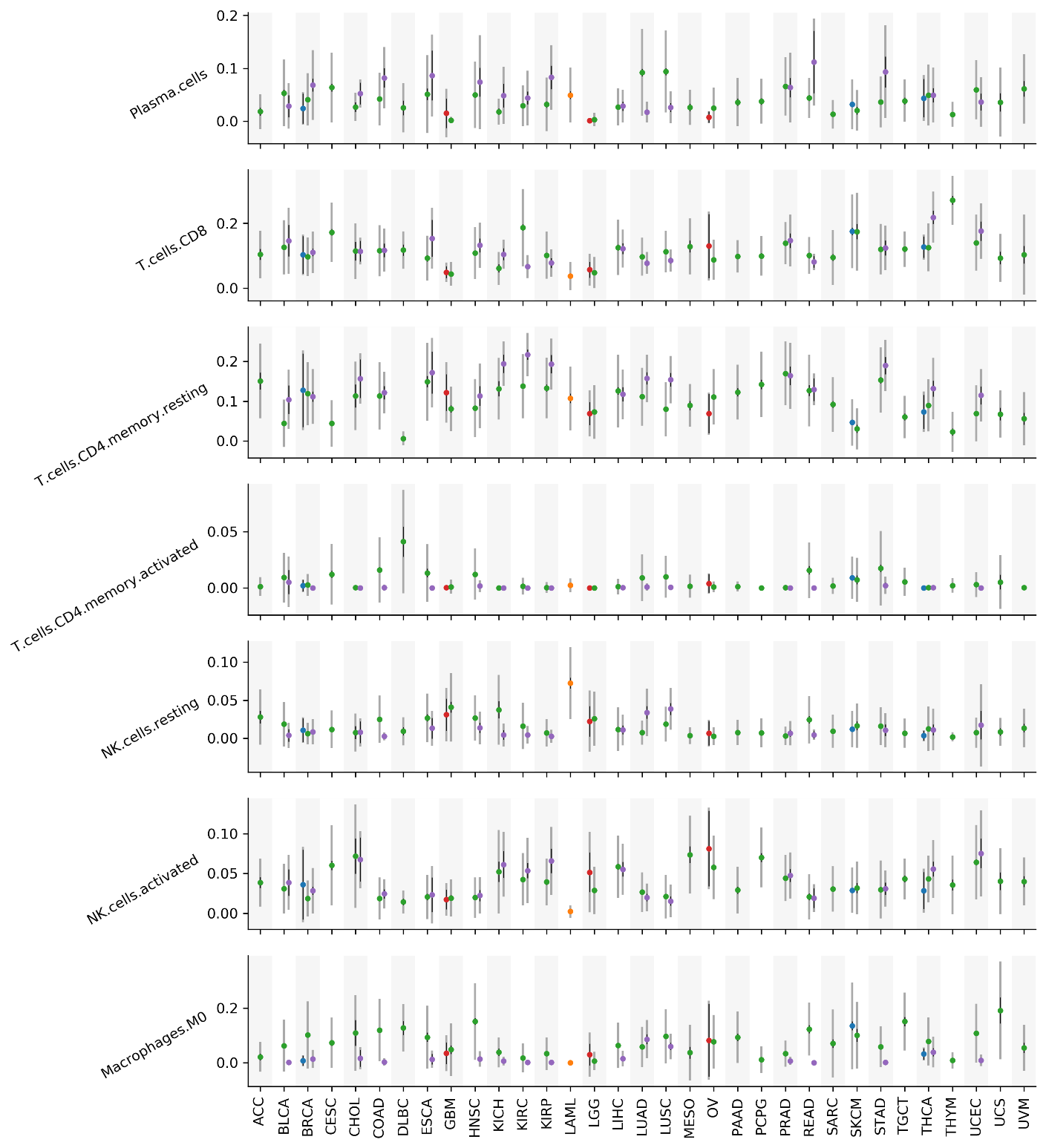


**Figure 5.** Mean, 95% confidence interval of mean, and standard deviation the abundance for selected immune cell types in the deconvolved TCGA data. Scheme is the same as Figure S4. Remaining cell types are shown in Figure S6.


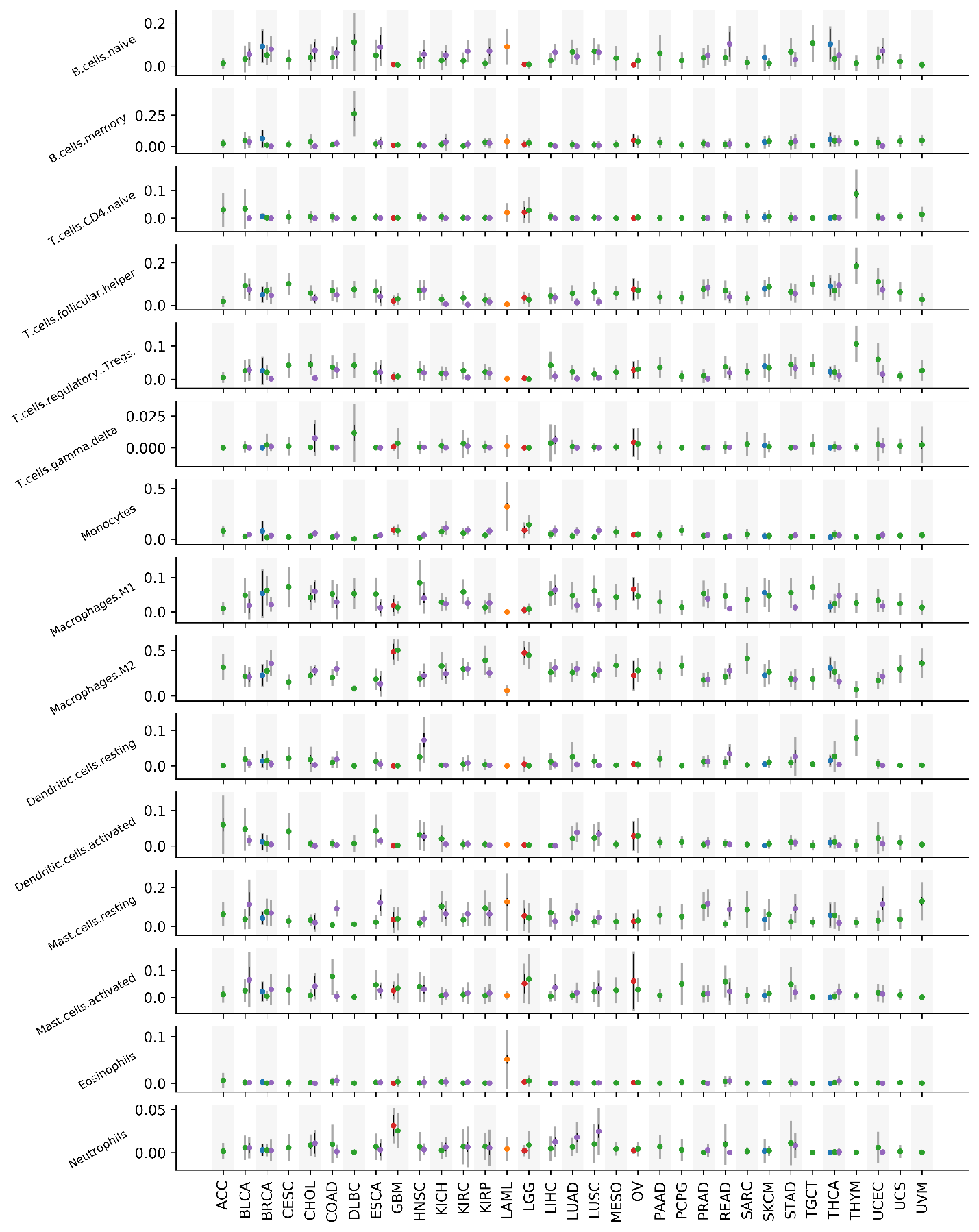


**Figure S6.** Mean, 95% confidence interval of mean, and standard deviation the abundance for remaining immune cell types in the deconvolved TCGA data. Scheme is the same as Figure S4 and S5. Other cell types are shown in Figure S5.


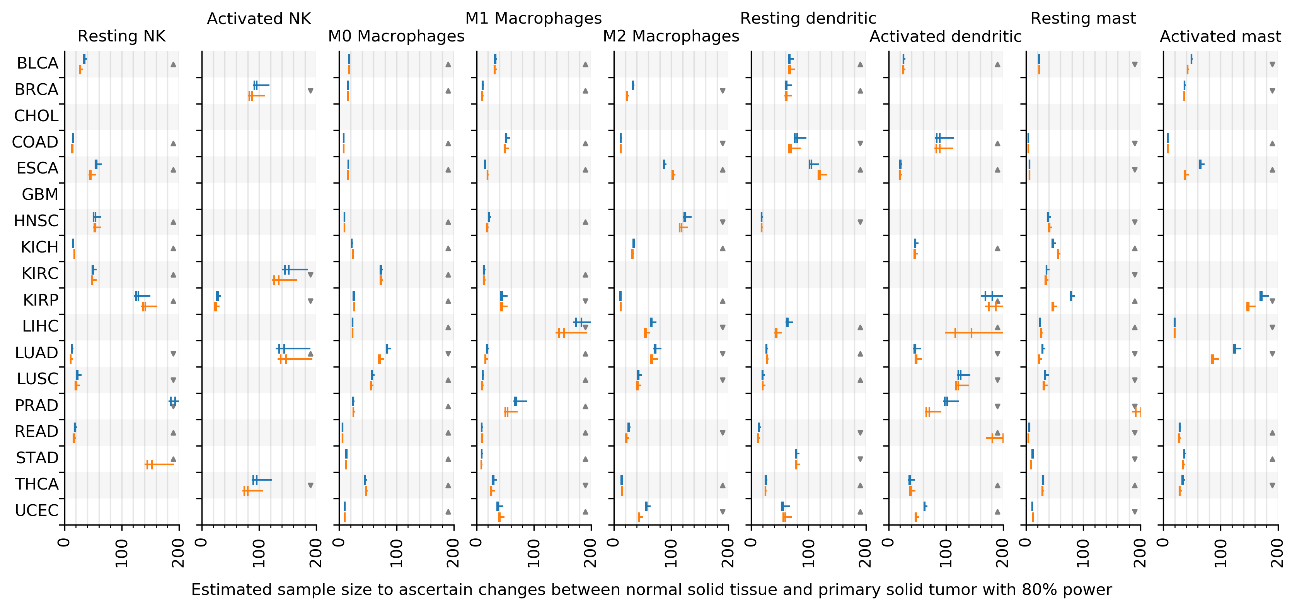

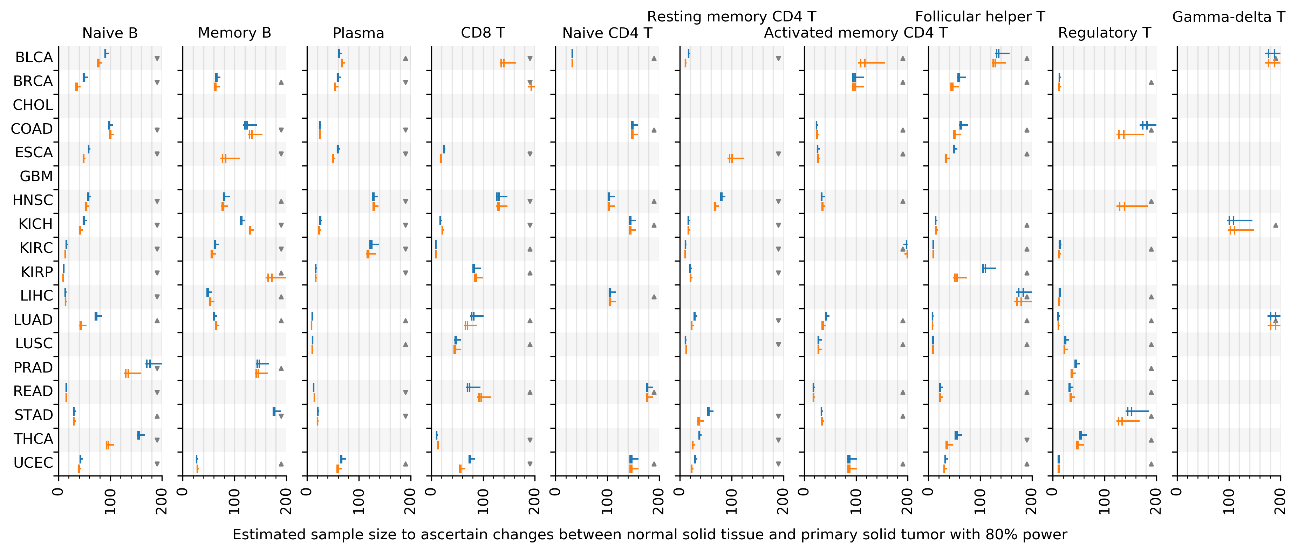

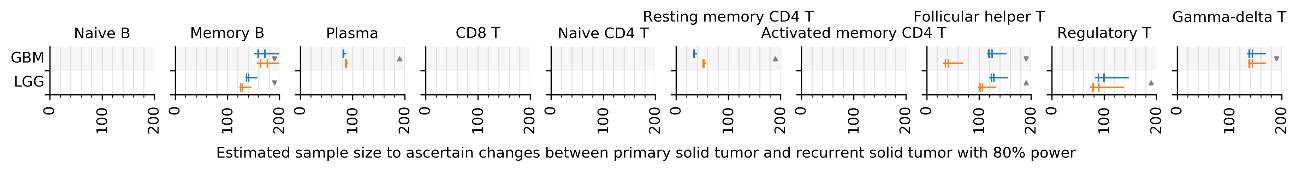

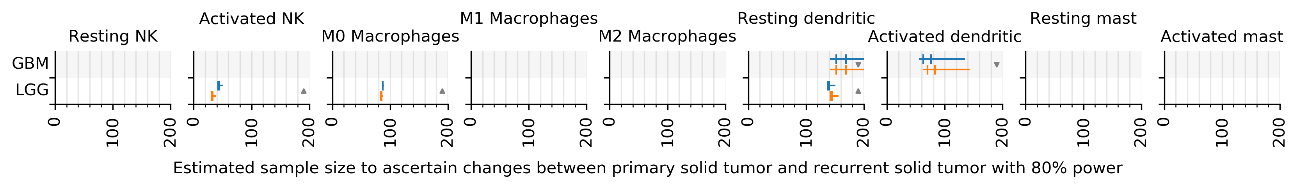

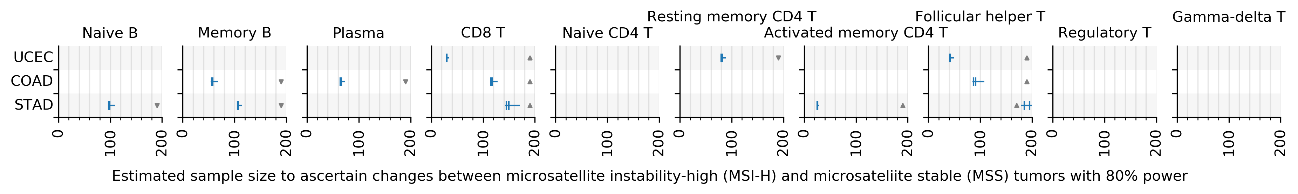

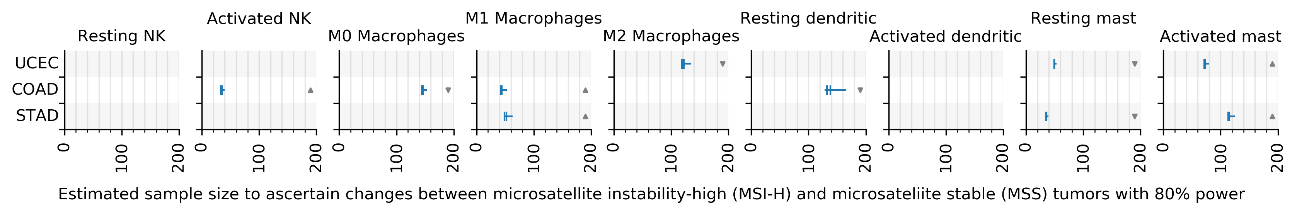


f

e

d

c

b

a

**Figure S7 related to Fig. 3.** Sample size estimation by Sensei. **a,b** Estimated sample size for primary tumor vs recurrent tumor. More cell types are shown to supplement Fig. 3a. Estimations for unpaired test and paired test are shown in blue and yellow, respectively. Estimations are for infinite (left end of a whisker), 1,000 (left bar), 384 (right bar), and 100 (right end of a whisker) cells. Fewer cells require more samples to ascertain an effect. **c,d** Sample size estimation for normal tissue and tumor. More cell types are shown to supplement Fig. 3b. **e,f** Sample size estimation for MSI-H and MSS tumor. More cell types are shown to supplement Fig. 3c. Sample sizes over 200 are omitted.


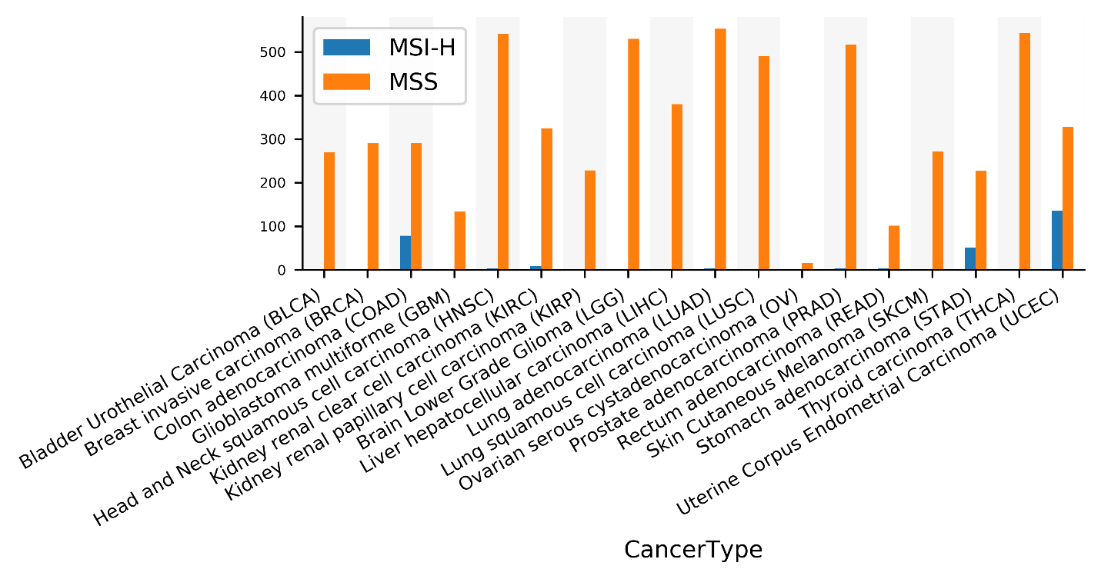


a


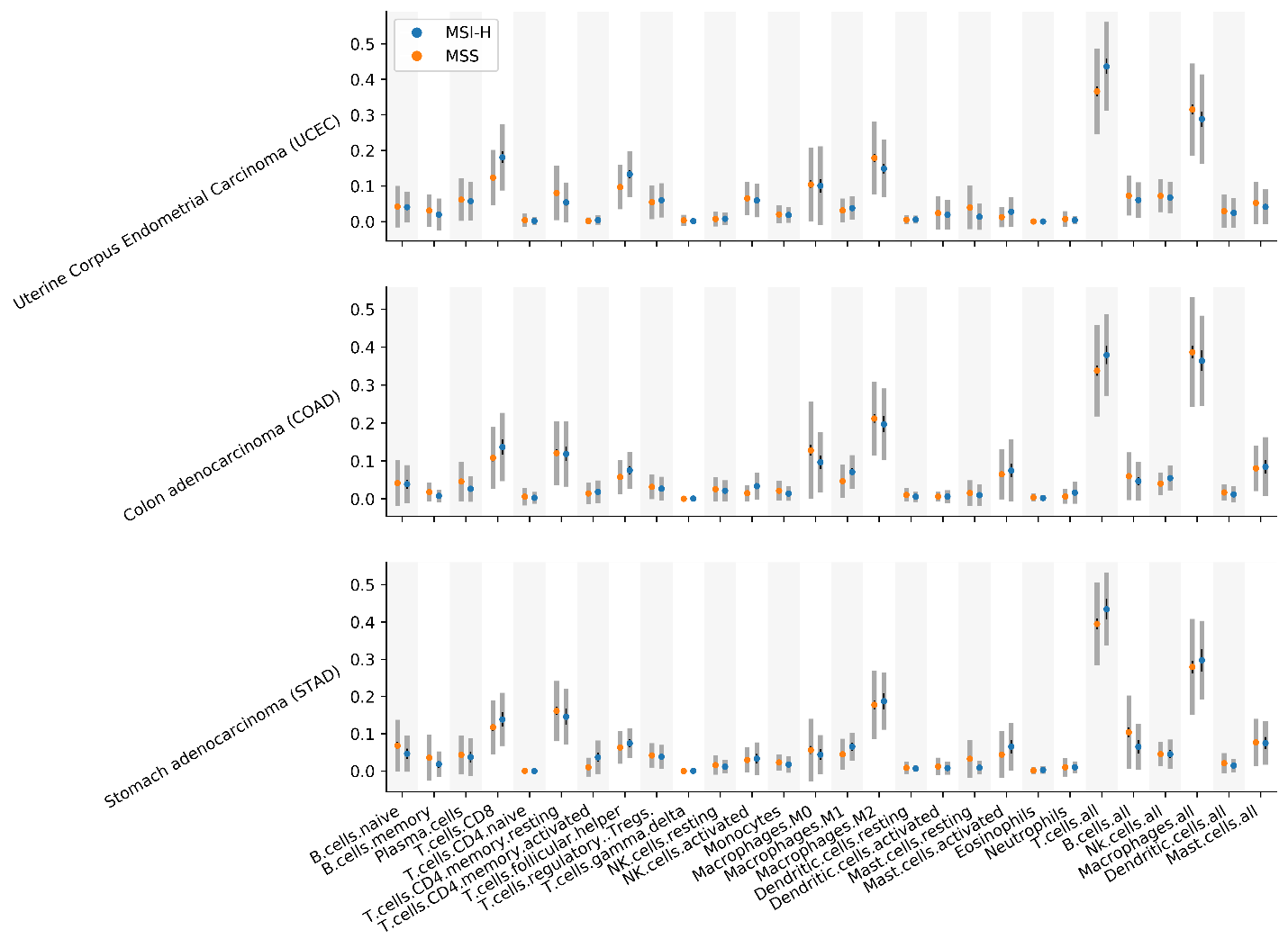


b

**Figure S8. a** Number of MSI-H and MSS samples in each cancer type. **b** Immune cell type abundance in MSI-H and MSS tumor samples in UCEC, COAD, and STAD. Sample mean (dot), sample standard deviation (thick gray line spans a total of two times the standard deviation), and 95% confidence interval (think black line) of sample mean are shown.

| 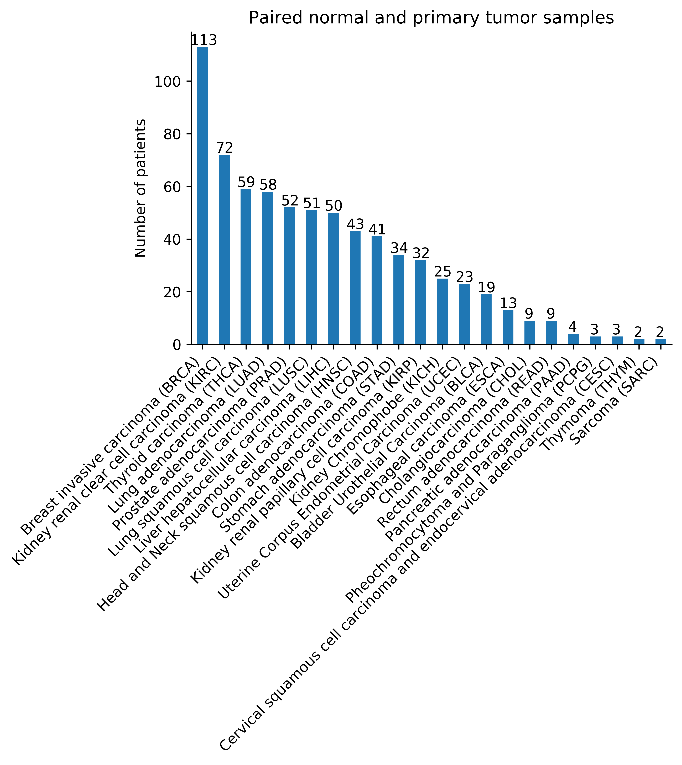  a | 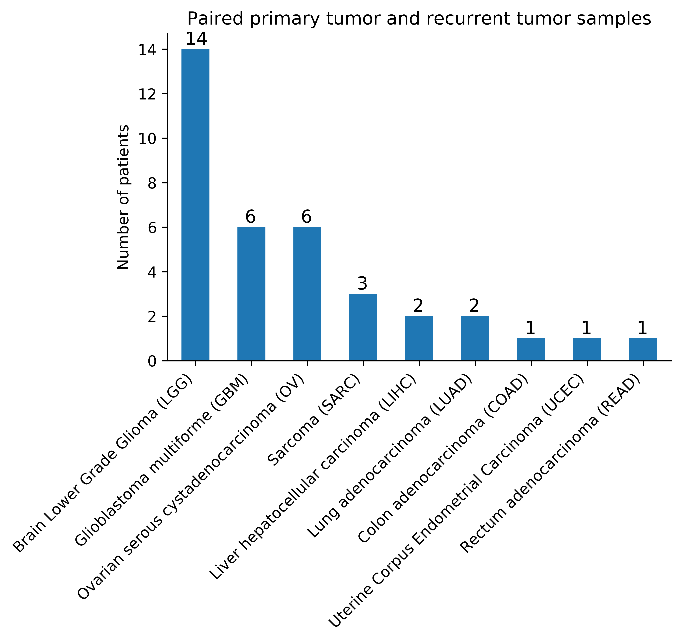  b |
| --- | --- |
| 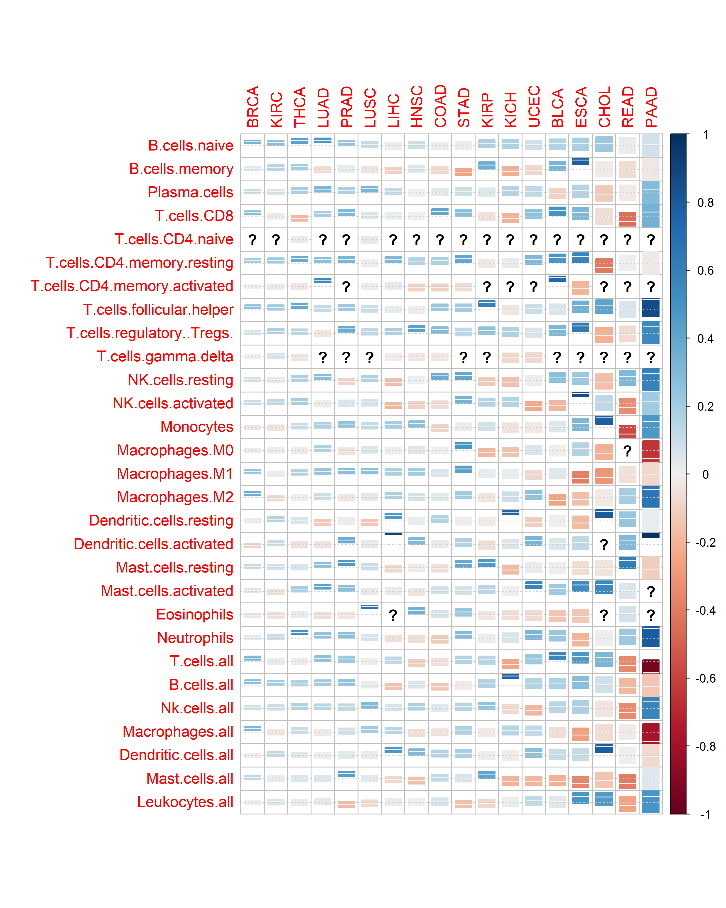  c | 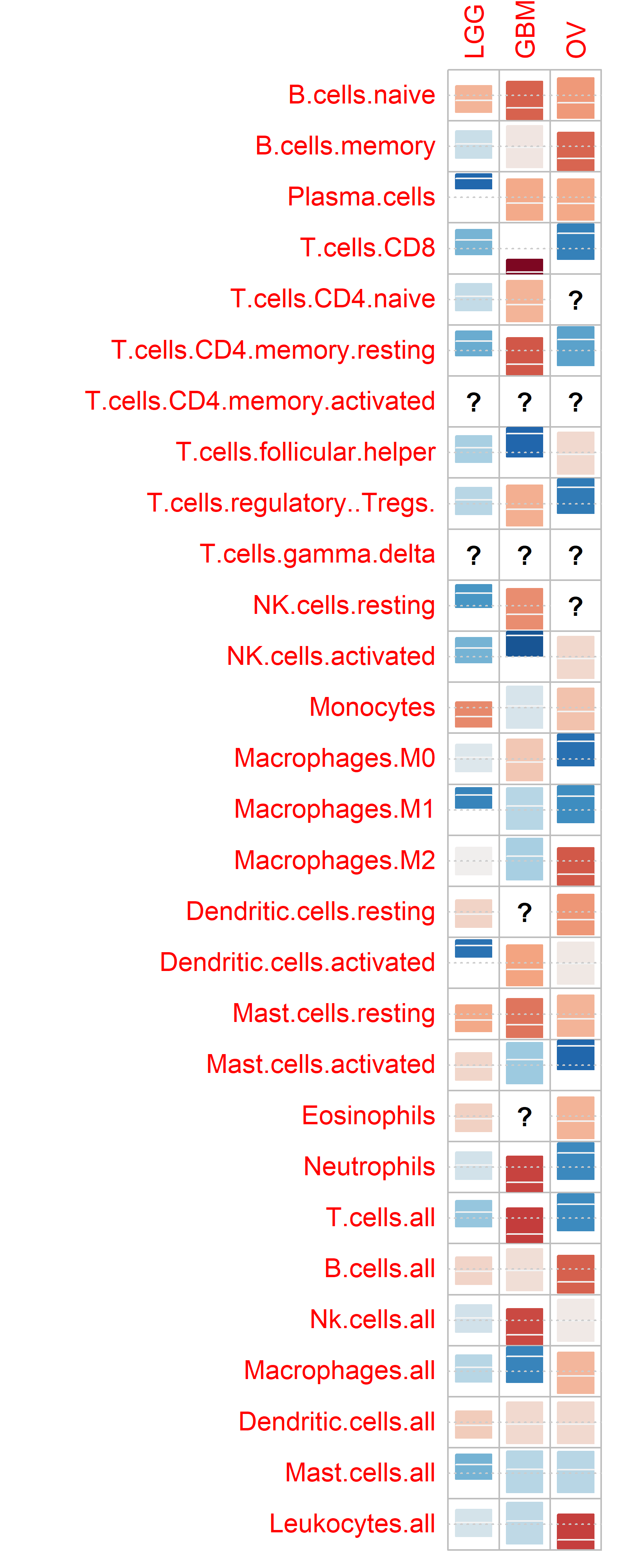  d |
| **Figure S9.** Correlation of cell type abundances in paired samples in cancer data. **a** Number of patients with paired normal and primary tumor samples in each cancer type in the deconvolved TCGA dataset. **b** Number of patients with paired primary tumor and recurrent tumor samples in each cancer type. Cancer types with no paired samples do not show. **c** Correlation (center line/color: correlation; rectangle: 95% confidence interval; question marks: insufficient unique datapoint for calculating the correlation) of immune cell type abundances between paired normal and primary tumor samples in each cancer type. Cancer types with less than 4 patients are omitted. **d** Correlation of immune cell type abundances between paired primary tumor and recurrent tumor samples in each cancer type. | |

| 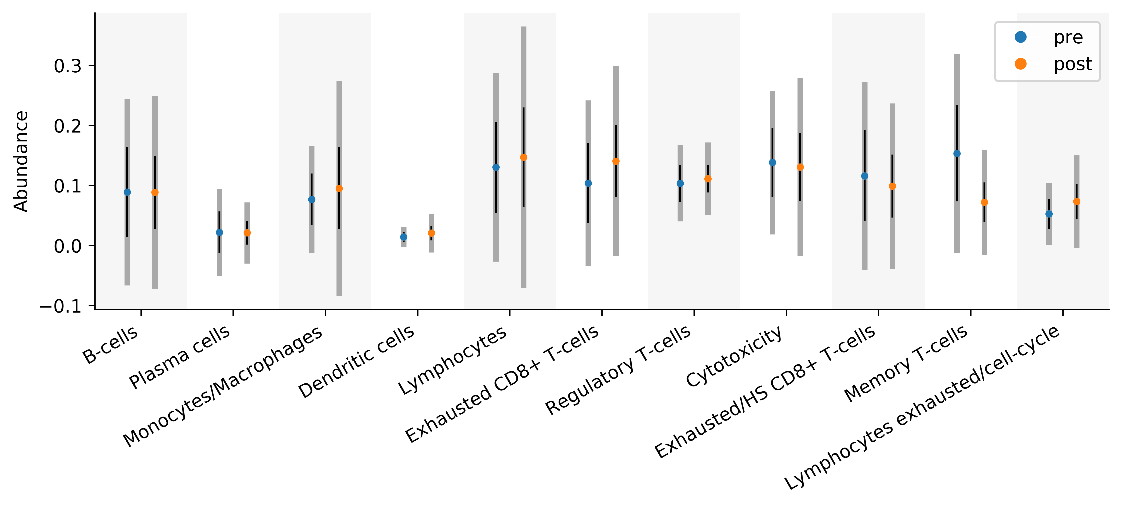  a | 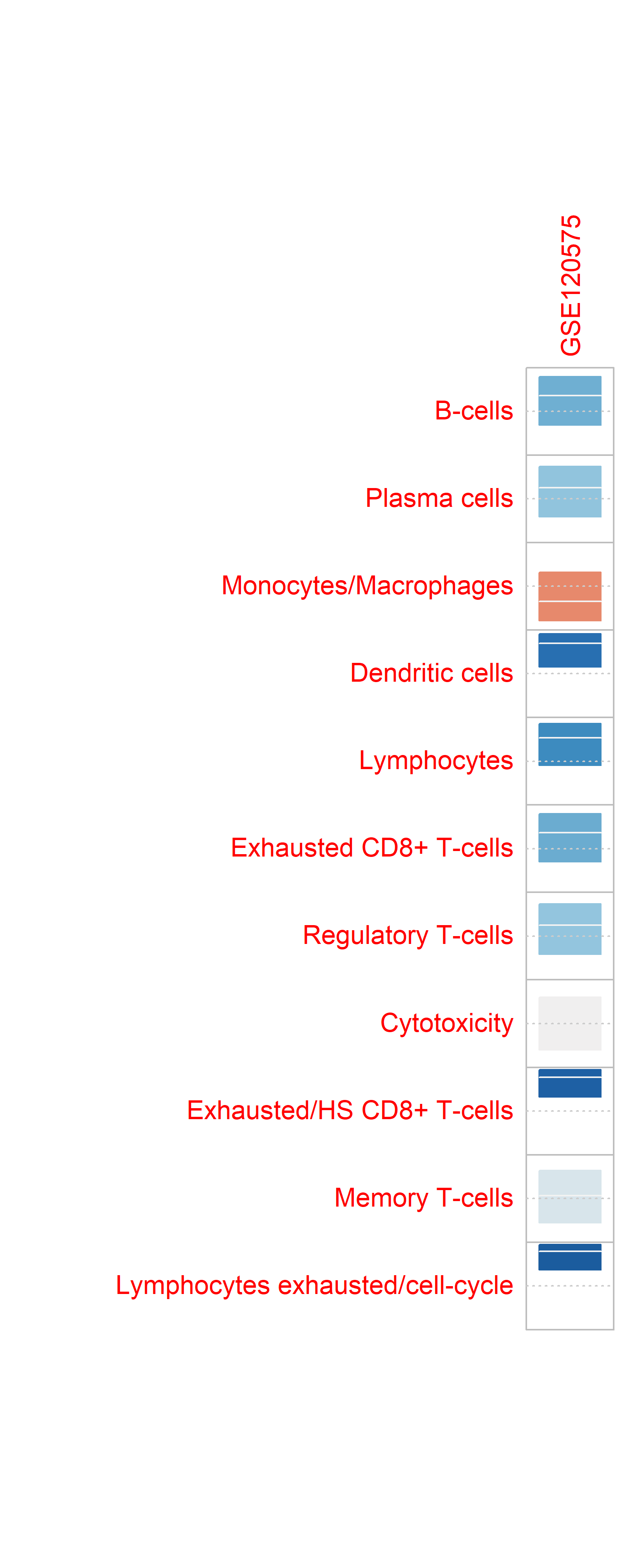  b |
| --- | --- |
| **Figure S10.** **a** Mean (dot), 95% confidence interval of mean (thin black line), and standard deviation (wide gray line) of the abundance of each major immune cell types in the metastatic melanoma data. **b** Correlation of immune cell types from pre- and post-treatment samples from metastatic melanoma patients. | |

|   Error (L1 distance of CDF)  Standard deviation $\sigma$  $\mu$  a | 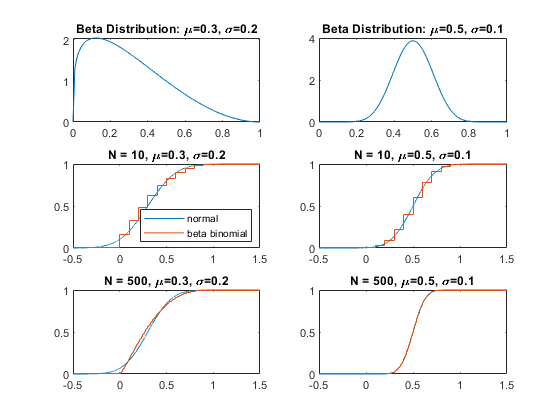  CDF  Density  CDF  b |
| --- | --- |
| **Figure S11.** Comparison of the scaled beta-binomial distribution and the normal estimation. **a** L1 distance between the scaled beta-binomial distribution and normal distribution. Subpanel: number of cells. Color: mean; X-axis: standard deviation; Y-axis: L1 distance between cumulative distribution function (CDF) of a scaled beta binomial distribution and its normal approximation. **b** Four representative cases. First row: corresponding scaled beta-binomial distribution. Second row and third row: CDF for four cases. | |

## Supplementary Note 1: Validation of normal distribution

Skewness and kurtosis are standard measures for asymmetry and extreme values in a sample, respectively. Sample drawn from a normal distribution have skewness close to 0, and kurtosis also close to 0 (mesokurtic). Lower or higher skewness indicates left or right skew. Sample with lower or higher kurtosis are termed platykurtic or leptokurtic.

Because normality is an important assumption of the t-test, we verify it by calculating the skewness and kurtosis (Methods) of cell type abundances in TCGA data. Thorsson et al. deconvolved bulk RNA-seq data from TCGA data and obtained the proportion of immune cells [2]. Across 33 cancer types, available are 22 immune cell types, which we further grouped into 6 major types (T-cells, B-cells, NK cells, Macrophages, Dendritic cells, Mast cells). We calculated the skewness of each cell type in each sample, including normal tissues, primary tumors, and recurrent tumors. We found 90.5% of them between -3 and 3, and 78.0% between -2 and 2, indicating relatively good normality [28]. When considering the subtypes, as the abundances are closer to 0, distributions are naturally right-skewed. Nevertheless, 68.9% of the skewness is between -3 and 3, and 51.5% are between -2 and 2. The kurtosis, which is known to have less influence on the accuracy of the t-test [27], is also found to be within a reasonable range for most of the types. In specific, 87.5% and 66.3% of them are within -1 and 10 for major types and subtypes, respectively [28]. Overall, the t-test is proper for most of the immune cell types in most cancer types.

## Supplementary Note 2: User Manual for Sensei

We created a standalone web application using HTML and JavaScript. As Fig. 1 and Supplementary Figure 1 show, Sensei asks users to input a set of parameters for each group $i\in\{0,1\}$. Firstly, Sensei needs ranges of sample sizes $M_{i}$ for each group, which may factor in the financial feasibility and number of participants that can be gathered practically. Secondly, the number of cells in each single-cell sample, $N_{i}$, is needed to account for the variation introduced by limited/finite number of cells. Then, Sensei asks for the proportion of the cell type of interest in each group. Because cell type proportions vary among participants (in the same group) and may further be confounded by abovementioned technical variations, the proportion is not a deterministic value, but a random variable drawn from a beta distribution, which can be set by providing the mean $\mu$ and variance $\sigma$, or if preferred the standard parameters $\left( a_{i},b_{i} \right)$ for a beta distribution. These parameters may be obtained by surveying the literature, or by performing a pilot study, and a general guideline generated from TCGA is provided below. The graph at the top-right corner visualizes the beta distribution to double check if the distribution is satisfying. Finally, the user needs to set a false positive rate $\alpha$for t-test, usually 0.05 or 0.01, and a desired false negative rate $\beta$. For example, in Fig. 1g,h, Sensei generates a table of $\beta$ for $M_{0},M_{1}=8\sim12,$ $N_{0},N_{1}=1,000$, $\mu_{0}=0.3, \sigma_{0}=0.15, \mu_{1}=0.5, \sigma_{1}=0.1$, and false positive rate for t-test $\alpha=0.05$. A quick step-by-step guide is available at the webpage.

## Supplementary Note 3: Uncertainty in the estimated sample size

Uncertainty in the prior knowledge will propagate the estimated sample size. To reflect the uncertainty, a “confidence interval” can be constructed. When variances are fixed, the sample size is largely depending on the difference of the mean proportions of two groups. Thus, we calculate the confidence interval of the difference of the means. Using the Welch-Satterthwaite method, for two independent samples {$x_{0i}\}, i=1\ldots n_{0}$ and {$x_{1i}\}, i=1\ldots n_{1}$, the confidence interval $[\Delta\bar{x}_{L},\Delta\bar{x}_{U}]$ for $\Delta\bar{x}=\bar{x}_{1}-\bar{x}_{0}$ is constructed as

$$\begin{aligned} \Delta\bar{x}_{L},\Delta\bar{x}_{U}=\Delta\bar{x}\pm t_{1-\alpha/2,\nu}\sqrt{\frac{s_{0}^{2}}{n_{0}}+\frac{s_{2}^{2}}{n_{1}}},\#\left( SEQ Equation \backslash* ARABIC 1 \right) \end{aligned}$$

where $(1-\alpha)$ is the confidence level and the degree of freedom, and

$$\begin{aligned} \nu=\frac{\left( \frac{s_{0}^{2}}{n_{0}}+\frac{s_{1}^{2}}{n_{1}} \right)^{2}}{\frac{s_{0}^{4}}{n_{0}^{2}(n_{0}-1)}+\frac{s_{1}^{4}}{n_{1}^{2}(n_{1}-1)}}.\#\left( SEQ Equation \backslash* ARABIC 2 \right) \end{aligned}$$

We override the difference in the last step of sample size estimation. Note that if any of $\Delta\bar{x}$, $\Delta\bar{x}_{L}$, and $\Delta\bar{x}_{U}$ are of different signs, we consider the results unreliable.
